# Real-time ultrasound-guided pigtail catheter chest drain for complicated parapneumonic effusion and empyema in children – 16-year, single-centre experience of radiologically placed drains

**DOI:** 10.1007/s00247-018-4171-3

**Published:** 2018-06-27

**Authors:** Megan R. Lewis, Thomas A. Micic, Iolo J. M. Doull, Alison Evans

**Affiliations:** 1 Department of Postgraduate Medical and Dental Education at Cardiff University, Heath Park Way, Cardiff, UK CF14 4YU; 2 Department of Paediatric Radiology, Children’s Hospital for Wales, Heath Park, Cardiff, UK CF14 4XW; 3 Department of Paediatric Respiratory Medicine, Children’s Hospital for Wales, Cardiff, UK CF14 4XW

**Keywords:** Children, Drain, Empyema, Image-guided therapy, Parapneumonic effusion, Pigtail catheter, Pleura, Pneumonia, Ultrasound

## Abstract

**Background:**

Chest tube drainage with fibrinolytics is a cost-effective treatment option for parapneumonic effusion and empyema in children. Although the additional use of ultrasound (US) guidance is recommended, this is rarely performed in real time to direct drain insertion.

**Objective:**

To evaluate the effectiveness and safety of real-time US-guided, radiologically placed chest drains at a tertiary university hospital.

**Materials and methods:**

This was a retrospective review over a 16-year period of all children with parapneumonic effusion or empyema undergoing percutaneous US-guided drainage at our centre.

**Results:**

Three hundred and three drains were placed in 285 patients. Treatment was successful in 93% of patients after a single drain (98.2% success with 2 or 3 drains). Five children had peri-insertion complications, but none was significant. The success rate improved with experience. Although five patients required surgical intervention, all children treated since 2012 were successfully treated with single-tube drainage only and none has required surgery.

**Conclusion:**

Our technique for inserting small-bore (≤8.5 F) catheter drains under US guidance is effective and appears to be a safe procedure for first-line management of complicated parapneumonic effusion and empyema.

## Introduction

Parapneumonic effusion and empyema are well-recognised complications of pneumonia in children, resulting in significant morbidity and hospital admission. Although the incidence in the developing world appears to be increasing, outcomes following treatment are excellent, long-term complications are rare and mortality rates almost negligible [1, 2]. Parapneumonic effusions may progress from a simple, non-septated effusion (exudative phase/stage 1) through the more complex fibropurulent phase with fibrin strands and loculations (stage 2) to an organised multiloculated empyema with thick pleural rind (stage 3) [2].

Although antibiotics alone may suffice in some patients, those with large or more complicated effusions and those not responding to medical management frequently require drainage. The optimal treatment is contentious, but there is agreement that either chest tube drainage with intrapleural fibrinolytics or video-assisted thoracoscopic surgery are the treatments of choice [3]. There is a wealth of literature on adults, and more recently a number of randomized controlled trials in children, demonstrating both options to be safe and successful, with little outcome advantage of one procedure over another [4–7]. Ultimately, management usually depends on local clinical preference, the availability of different techniques and operator expertise.

In 2001, we published a short pilot study [8] that compared primary thoracotomy, surgically placed stiff drains with instillation of urokinase and small-bore radiologically placed drains with instillation of urokinase. We found a significant improvement in outcome measures for the use of small-bore tubes over large-bore surgically placed drains, which were better tolerated, reduced discomfort and offered cosmetic advantages. Outcomes for radiologically placed drains and thoracotomy groups were similar. Consequently, since that time, we have preferred to use real-time ultrasound (US)-guided small-bore pigtail catheter drainage with instillation of urokinase instead of other treatment options (video-assisted thoracoscopic surgery was not available at our centre).

In this retrospective review, we describe our technique and evaluate the efficacy and safety of our single tertiary paediatric centre experience over a 16-year period. We discuss our results in light of recent literature, changing epidemiology and complication rates.

## Materials and methods

Our institution serves a population of approximately 2.5 million people in South and Mid Wales, United Kingdom. Since our original report [8], all complicated paediatric pneumonias have been referred to the paediatric respiratory team at the Children’s Hospital for Wales, either admitted directly or referred from outlying hospitals. We identified, through paediatric respiratory and radiology databases, all children admitted with pneumonia and parapneumonic effusion/empyema requiring drain insertion between January 1, 2001, and December 31, 2016. We excluded those with drains inserted without direct US guidance by paediatric intensive care unit clinicians, surgically sited drains and drains for noninfectious causes including malignant effusions. To comply with ethical standards, we went through the National Health Service Research Ethics Committee pathway. Formal approval was not required.

We used a pragmatic approach to assess patients using chest radiographs and US. computed tomography (CT) was not routinely performed. The decision to insert a drain was based on effusion size, evidence of complicated parapneumonic effusion/empyema (increased echogenicity, strands, loculations or pleural rind), worsening of clinical parameters and/or failure to respond to 48 h of appropriate parenteral antibiotics (intravenous co-amoxiclav or cefotaxime plus intravenous clindamycin). Whether to insert a drain and the timing of drain insertion was based on a multidisciplinary discussion between the paediatric respiratory and radiology teams.

Drains were usually inserted under general anaesthesia in the operating theatre if the patient was younger than 4 years of age. In cooperative older children (4 years or older), drains were inserted in the radiology department under conscious sedation (intravenous pethidine and midazolam administered by the paediatric medical team) with appropriate monitoring. Local anaesthesia was routinely instilled at the drain site. A small-bore pigtail catheter (Dawson-Muller, Cook, UK) was inserted using a single-step trocar method under direct US guidance by one of our four consultant paediatric radiologists or a senior specialty training registrar under supervision. The optimal position for the drain site was determined by US, usually the largest component, which was on occasion outside the recognised safe triangle (distinguished by the lateral border of the pectoralis major anteriorly, the lateral border of the latissimus dorsi posteriorly, and inferiorly by a horizontal line from the nipple, commonly the 5th intercostal space) used for non-guided procedures. The drain was secured to the skin using a prefabricated adhesive device and dressing rather than suturing to the skin.

Immediately following drain insertion, pleural fluid was aspirated (maximum 10 ml/kg) to allow for expansion of the underlying lung. A sample was also sent for microscopy and culture. Subsequently, urokinase was instilled via the drain (40,000 IU diluted in 40 ml 0.9% saline if >1 year of age and 10,000 IU in 10 ml saline if <1 year of age), the drain clamped for a dwell time of 4 h during which time mobilization was encouraged to aid dispersion, followed by 8 h of drainage with negative pressure suction at 10–20 cm H_2_O. The cycle of instillation and drainage was repeated as clinically indicated, usually for six cycles.

Chest radiographs were not routinely performed immediately post-procedure, only if there was clinical indication. Drain removal was based on improving clinical signs and symptoms, normalization of temperature, daily drain volume, and US and radiographic appearances. If there was failure to improve clinically, further management was guided with US and/or CT along with multidisciplinary discussion regarding a second procedure/surgical intervention.

Patient demographic data, including patient’s gender, age at presentation and preexisting or underlying medical conditions, were abstracted from radiology records, patient medical records and laboratory databases. Data on the procedure’s laterality, drain size, duration of drain in situ, duration of stay post drain insertion and total hospital stay were collected. Patients undergoing a CT scan as part of their management were reviewed and microbiology results abstracted.

Complications were reviewed, including those directly related to the procedure itself and those developing subsequently, including misplacement, the need for repositioning and complications resulting from the underlying disease process. The primary outcome measure was successful clinical treatment by drain alone – failure was defined by the need for a second procedure including a second pigtail drain, surgically placed drain or thoracotomy. Secondary outcome measures were days of drain in situ, days from drain insertion to discharge and complications. Analysis of time trends (4-year epochs) used chi-square test.

## Results

Over the 16-year period, 303 drains were inserted into 285 children (112 female; median age: 5.2 years, age range: 0.5–15.9 years) for complicated parapneumonic effusion or empyema. Sixty-six children (23.2%) were admitted directly to our institution, and 219 (76.8%) were from local hospitals.

The majority of drains (202/303; 66%) were placed under general anaesthesia, with 101 (33%) patients placed under sedation. The youngest patient undergoing the procedure under sedation was 9 months. There was equal distribution of drains with 153 (50.5%) drains being inserted on the right and 150 (49.5%) on the left. The most common drain size used was 8.5 Fr (86.5%), the remainder were 7 Fr (12.9%) except for an 8-month-old child who received a short 6.3-Fr catheter and an 11-year-old patient who received a 10-Fr tube (their third drain).

Two hundred sixty-five children (93%) were successfully treated with a single drain insertion. Twenty children required further intervention, 17 during the initial admission and 3 following readmission. During the initial admission, 9 of 17 had a second drain inserted for treatment failure, 2 for contralateral pleural collections, 2 because the original drain became dislodged/fell out and 1 drain was inserted in the intensive care unit without US guidance. Another three children proceeded to thoracotomy.

Three children were discharged and then readmitted without resolution of the parapneumonic effusion/empyema. One child was readmitted 5 weeks post initial drain insertion with re-accumulation and a bronchopleural fistula and proceeded to thoracotomy; one child required two drains during the initial admission and was then readmitted 2 weeks later with a further re-accumulation requiring a third drain; and one child with Down syndrome was readmitted 5 weeks post initial drain insertion and required two further drains. Thus, all the patients who required three drains were admitted twice.

Thirty-one children (10.9%) had a preexisting medical condition, several with multiple comorbidities. Nine had longstanding developmental, congenital or chromosomal abnormalities (including trisomy 21, Sanfilippo syndrome and a variety of neuromuscular disorders). Six had respiratory conditions – secondary to prematurity (2), asthma (3) and diaphragmatic paralysis (1). Three had significant renal disturbance, one resulting from haemolytic uraemic syndrome. Four were recovering from abdominal surgery (three post appendicectomy and one post inguinal hernia repair) and one from post abdominal trauma. Two were significantly obese and one was anaemic. Five had had recent varicella infection, one child was HIV positive and one patient had recently received treatment for a systemic streptococcal infection (scarlet fever).

**Fig. 1:**
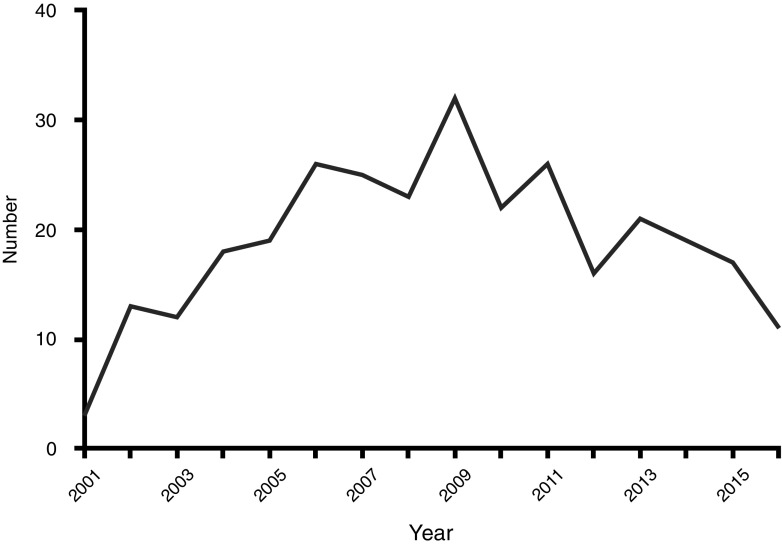
The number of children with empyema requiring percutaneous drainage 2001–2016

There was a noticeable increase in children admitted from 2001 to 2009, reaching a peak in 2009 and then decreasing over the years 2010 to 2016 (Fig. 1). The mean (range) duration of the drain in situ was 5.5 (1–26) days, while the mean (range) length of stay was 9.0 (1–40) days. Although the mean duration of the drain in situ was similar in those with pre-morbid conditions when compared to those without (6.0 vs. 5.5 days, 95% confidence interval of the difference: -0.9-1.8; *P*=0.5), their length of stay was significantly longer (13.1 vs. 8.5 days, 95% confidence interval of the difference: 7.8–1.3; *P*=0.007).

**Fig. 2:**
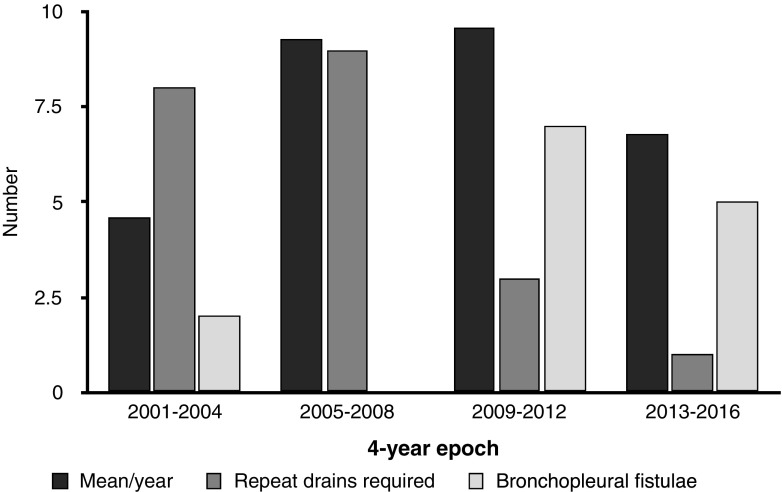
The mean number of drains inserted, the total number of patients requiring a further drain and the number of children with secondary bronchopleural fistula by 4-year epochs

The frequency of repeat drain insertion and/or surgical interventions has decreased significantly over the 16-year period (chi-square for quartiles=17.36, *P*<0.01) (Fig. 2). No child has required a thoracotomy or surgical drain since 2011, and none has required a second drain since 2012.

Five children had periprocedural complications all related to incorrect, suboptimal or failed siting. In two cases, the operator did not believe that siting was correct during the procedure, and placed a further drain at the same intervention (one of these developed a small pneumothorax). One case did not drain well and there was concern that the tube may just breach the lung edge on CT; it was consequently removed and the patient recovered conservatively. The other two patients had incorrect siting of their drains, one into the lung fissure and the other into the subcutaneous tissues (both diagnosed on CT). One was conservatively managed and the other had a larger bore drain sited surgically. Both patients were significantly obese. There were four minor periprocedure complications, two with small pneumothoraces and two minor bleeding, none of which required further intervention.

The drain fell out in four children (two required re-siting), and four children developed pathology on the contralateral side - one spontaneous pneumothorax and three contralateral pleural collections, two of which required a second drain.

Fourteen children developed a bronchopleural fistula: 11 cases were managed conservatively, 1 had a pleural aspiration and 2 had further pleural drains, 1 inserted by the pediatric intensive care unit (PICU) team. Bronchopleural fistulae were significantly more frequent in the second half of the study period (Fig. 2) compared to the first (3 of 135 cases vs. 13 of 150, chi-square 5.1, *P*=0.03). The mean duration of the drain in situ (11.9 vs. 5.2 days, 95% confidence interval of the difference: 10.6–2.9; *P*=0.002) and the length of stay (18.0 vs. 8.5 days, 95% confidence interval of the difference: 15.0–3.9; *P*=0.003) were both significantly longer in those with bronchopleural fistula. Those with comorbidities were at no increased risk of developing bronchopleural fistula (chi-square 0.4, *P*=0.6).

Thirty-four CT scans were performed in 30 patients to aid diagnosis in 6 patients and to evaluate possible complication in the other 24 cases. Four patients underwent CT post discharge for suspected underlying congenital lung lesion.

Potential causative organisms were identified in only 53 children (18%), the most common being *Streptococcus pneumoniae* (23) followed by group A Streptococcus (12) and *Staphylococcus aureus* (7). Other organisms identified included mycoplasma, *Haemophilus influenzae* and *Mycobacterium tuberculosis*.

## Discussion

Parapneumonic effusion and empyema are relatively uncommon in children; however, the incidence has been reported to be increasing, particularly in younger patients [1, 2, 9]. It is estimated that between 1% and 12% of community-acquired pneumonias are complicated by parapneumonic effusion/empyema, and up to 40% in those admitted to hospital [10].

The aetiology, causative agents, risk factors, morbidity and mortality rates are completely different in adults [2], thus extrapolation from adult data is not appropriate. It is recommended that all children with complicated pneumonia, particularly those who may need drainage, are managed at a tertiary paediatric unit [2, 10, 11]. At our centre, treatment decisions are made collaboratively between the paediatric respiratory and radiology teams. Small effusions and patients with mild respiratory distress usually respond to antibiotics alone. Although many complex effusions will improve with conservative management alone, the treatment course will be longer [2].

Drainage of a large parapneumonic effusion or empyema is associated with decreased length of stay compared to antibiotics alone [2], and other treatment options include chest drain insertion with or without fibrinolytics and surgical options including video-assisted thoracoscopic surgery and thoracotomy. Compared to instilling saline through chest drains, instilling fibrinolytics is associated with increased pleural drainage [12] and decreased length of hospitalisation [13]. These benefits appear greater with the use of small-bore compared to large-bore chest drains [8, 13], and possibly with early intervention [14]. There is debate over the relative benefits of chest drain insertion with the instillation of fibrinolytics or video-assisted thoracoscopic surgery [3, 15], and individual patient management is often dependent upon local practices, physician preference and availability.

There are four prospective randomised controlled studies in children comparing video-assisted thoracoscopic surgery with chest drain insertion and instillation of intrapleural fibrinolytics [4–7]. Three of the four studies had very similar duration of chest drainage, length of hospitalisation and the need for thoracotomy between the two treatment regimens [4–6]. In one study, which is more difficult to interpret, the video-assisted thoracoscopic surgery patients had a significantly shorter duration of symptoms after intervention and a shorter hospital stay [7]. However, the patient group in this study had more advanced disease having presented later, the investigators used large-bore chest drains and the thoracotomy rate was much higher in both groups. In the three studies that assessed cost, chest drain with instillation of fibrinolytics was significantly less costly than video-assisted thoracoscopic surgery. In a national surveillance study in Germany examining the treatment of 645 children with parapneumonic effusion/empyema over nearly three years, less than a quarter required invasive intervention with only 89 receiving a chest drain with intrapleural fibrinolysis and 43 a surgical procedure (either video-assisted thoracoscopic surgery or an open thoracotomy). There were no significant differences in the length of stay between surgical and drain patients [16].

In the UK, drain insertion with instillation of fibrinolytics is more widely available than video-assisted thoracoscopic surgery. Children undergoing video-assisted thoracoscopic surgery will require general anaesthesia and usually CT prior to the procedure, both of which may be avoided with chest drain insertion [4, 17]. Currently, the American Paediatric Surgical Association says there is sufficient high-quality evidence to support the use of chest drain insertion and fibrinolysis as the primary treatment modality in childhood empyema, with video-assisted thoracoscopic surgery procedures being reserved for difficult cases where fibrinolysis has failed [18].

Thoracotomy is now rarely used as a primary treatment – in a recent United Kingdom survey [19], only 2% of patients underwent primary thoracotomy -- and is usually reserved as a rescue treatment. In our series, only four children (1.4%) proceeded to thoracotomy, all after the failure of drain insertion. It is our perception that with increasing experience, the frequency of thoracotomies has decreased and no child has required thoracotomy since 2011. The only indication for primary thoracotomy is probably for a very late presentation of a large organised empyema, where both fibrinolysis and video-assisted thoracoscopic surgery are less effective.

In line with current guidelines [2], we use chest radiography and US to assess the severity of pneumonia, size and appearance of the effusion, and any complications such as abscess formation or bronchopleural fistula. US is safe and noninvasive and is the most useful investigation. Loculations/septations and increased echogenicity within the collection correlate moderately with purulence, but even anechoic effusions may contain frank pus. Overall, no reliable clinical, radiologic or pleural fluid characteristic can determine outcome, the only factor reported to be predictive for failure being necrotising pneumonia identified on initial imaging [19, 20]. Although US cannot reliably establish the stage of collection, it can estimate the effusion size and echogenicity, whether fluid is free or loculated, assess pleural thickening and guide the best site for drainage [11].

CT has a limited role and is unnecessary for diagnosing or managing most cases. Apart from the radiation risks involved, it is inferior at identifying septations and rarely provides useful information before intervention that is not identified on US. It may be required in complicated cases, for assessing misplaced tubes or bronchopleural fistulae/abscesses or prior to surgery [18, 21, 22]. Our use of CT for diagnosis has decreased over time; only one child has required CT at admission in the last 9 years. The most common indication for its use is in evaluating bronchopleural fistulae; however, only 71.4% of our patients in this group were evaluated with CT, the remainder were managed with plain film imaging alone.

Our practice, in line with national guidelines [2, 11, 18], is to insert small-bore chest drains and instill a fibrinolytic. Options for fibrinolytic agents include streptokinase, urokinase or alteplase, although in animal models there is little difference between agents. We have used urokinase routinely since 1998, and our protocol accords with previous controlled studies [13] and guidelines [2, 11].

There has been much debate on the optimal tube size for drainage in children. There are no controlled studies in children, but our experience [8] and that of others [13] is that smaller drains are associated with shorter length of stay. In contrast, Petel et al. [23] reported significantly higher failure rates for 8.5-Fr pigtail catheters compared to large-bore surgical drains. However, their drains were not inserted under US guidance, and their failure rate (40%) is significantly higher than our experience or that of other investigators. Based on patient age and size, US appearance of the collection and operator preference, we routinely insert 7-Fr drains, or 8.5-Fr drains for children older than 2 years. The drain is secured using a prefabricated adhesive device and dressing rather than suturing to the skin, which provides reliable fixation that is comfortable for the patient and results in a noticeably smaller scar. In our experience, small-bore tubes are less traumatic to insert, and allow increased patient mobility potentially improving dispersion of fibrinolytics within the pleural space. Only 4 of 303 drains fell out (1.3%) with the fixation system compared to rates as high as 13% in other series [4].

Drain insertion should comply with local and national guidelines, with a limited number of personnel performing the procedure, and should, if possible, be performed within normal working hours [24]. We have a small cohort of paediatric radiologists trained to perform this procedure and the majority of cases occur during working hours unless the clinical condition requires more urgent intervention. The procedure is well tolerated under sedation in older children, and is logistically more straightforward than general anaesthesia. The procedure is performed under general anaesthesia in young or uncooperative children or where there is respiratory compromise [2]. Only one patient required a short stay on the intensive care unit following general anaesthesia.

Ideally, drains should be inserted in the triangle of safety, but direct US guidance identifies the optimal site for insertion, which may be outside the recognised safe area. The patient may be positioned erect, supine or on their side for drain insertion.

Although it is recommended that drains should be inserted under US guidance, in practice it is unclear from many reports whether this is merely marking the optimal spot for drain insertion or real-time US-guided drain insertion. There are no controlled studies evaluating real-time US-guided chest drain insertion, but increased success rates have been reported [23, 25]. We believe that although marking the spot may be acceptable for large pleural collections in older children, real-time US guidance allows constant monitoring with complete visualisation of the needle tip, thus minimising the risk of drain malplacement or visceral or lung damage. Insertion complications such as bleeding and pneumothorax become quickly apparent and the drain position can be adjusted accordingly. The only disadvantage is that real-time US-guided drain insertion requires out-of-hours availability of appropriately trained staff, and may not be available in all units.

There are well-recognised complications of chest tube insertion (including small-bore catheters), mostly relating to visceral, lung and vascular injury [2, 24, 26, 27]. A National Patient Safety Association review over 3 years identified 12 deaths and 15 cases of serious harm resulting from chest drain injury to the heart, lungs and liver [28]. The Seldinger technique is recommended for drain insertion [2, 4–6, 11, 23] as is believed to be safer than using a trocar. However, the Medicines and Healthcare Regulatory Authority (United Kingdom) has highlighted a number of adverse events associated with the Seldinger technique, largely resulting from injury due to the stiff dilator. We routinely use a single-step pigtail catheter trocar system for the majority of our interventional procedures and have gained extensive experience in thoracic, abdominal and nephrostomy drains. The lack of body fat in children and generally low echogenicity of the pleural collection results in excellent visualisation of the drain system during insertion, even if there is significant loculation. There are significantly fewer steps than with the Seldinger technique and we find the procedure straightforward and quick to perform. Due to the superior visualisation of the trocar system using real-time US, collections as small as 1.5–2 cm can be safely accessed. Our complication rate was low and although there were cases of malplacement (significant obesity was the major risk), there were no serious visceral or lung injuries.

We defined success as not requiring either another drain or surgical intervention, and our success rate of 93% (arguably 94% if we exclude those who needed a second drain for contralateral effusions) compares favourably with previous studies that report between 83% and 93% success [4–6, 19]. Similarly, our mean length of stay at 9.0 days (even after including those with significant pre-morbid medical problems) is very similar to that in previous reports [4–6]. The exception is the study reported by Cobanoglu and colleagues [7] where 30% of the chest drain group progressed to video-assisted thoracoscopic surgery. However, many of these patients had more advanced and organised disease due to late referral patterns, and they used larger-bore drains. Of note is that 22% of those randomised to video-assisted thoracoscopic surgery required subsequent thoracotomy, supporting our belief that the role of thoracotomy is in late presenting organised empyemas.

Changing patterns in the epidemiology of pneumonia and empyema have been noted in recent years, with an increased incidence of more complicated pneumonias seen in children [1, 29, 30]. This not only results in increased morbidity, longer length of stay and overall health care costs, but inevitably increases the likelihood of complications including bronchopleural fistulae. Although long-term outcome following bronchopleural fistula is still excellent using chest drainage alone, the length of tube drainage and duration of hospital stay may be prolonged [2, 31]. McKee [30] reported a significant increase in the incidence of bronchopleural fistula in association with empyema, from 1% of cases in 2002–2007 to 33% of cases in 2008–2009, with an associated increase in median hospital stay.

Our data are consistent with these reports, and more recently we observed a significantly increased frequency of bronchopleural fistulae with resultant increased length of stay. It is postulated that changes in the epidemiology of causative pathogens with the introduction of pneumococcal conjugate vaccines have resulted in increased necrotising or cavitating pneumonias with abscess formation and bronchopleural fistulae [30, 31].

## Conclusion

Our 16-year experience demonstrates that our technique for inserting chest tube drains to manage parapneumonic effusion/empyema in children is a safe, effective procedure. We have a low rate of complication and a high rate of successful outcome, with both the duration of tube in situ and hospital stay post intervention comparable to published data. With experience, the need for a second drain or surgical intervention has decreased; however, we have noted increased complications relating to bronchopleural fistula since 2010, resulting in significantly increased hospital stays in these patients. We believe that the use of small-bore pigtail catheters and direct real-time US guidance has contributed to this success.
